# Multivariate mapping of brain pathology: a step forward with stumbling blocks

**DOI:** 10.1093/braincomms/fcae253

**Published:** 2024-07-30

**Authors:** Christoph Sperber, Roza Umarova

**Affiliations:** Department of Neurology, Inselspital, University Hospital Bern, University of Bern, 3010 Bern, Switzerland; Department of Neurology, Inselspital, University Hospital Bern, University of Bern, 3010 Bern, Switzerland

## Abstract

This scientific commentary refers to ‘Ground-truth validation of uni- and multivariate lesion inference approaches’, by Zavaglia *et al*. (https://doi.org/10.1093/braincomms/fcae251).

This scientific commentary refers to ‘Ground-truth validation of uni- and multivariate lesion inference approaches’, by Zavaglia *et al*. (https://doi.org/10.1093/braincomms/fcae251).

Statistical parametric mapping was the dominant method of brain mapping for many years. It assesses the relationship between each feature in a brain image (e.g. the signal in a voxel, region or fibre tract) and a target variable (e.g. a cognitive function or neurological deficit) with an independent univariate statistical test. Implementation and interpretation of such analysis are relatively easy and intuitive. However, independent univariate models have a significant limitation: they cannot represent the complex, multidimensional, nonlinear and interacting phenomena that characterize the functional architecture of the human brain.^1^

The recent work by Zavaglia *et al*.^2^ joins a collection of works that have addressed this issue in stroke lesion-deficit inference with a simulation strategy.^3-6^ A design in which the neural correlates of a simulated deficit were defined by the researchers themselves allowed an objective evaluation of lesion-deficit inference. They illustrated the shortcomings of univariate inference that produced highly unspecific results. On the other hand, two multivariate inference techniques, based on machine learning and game theory, achieved nearly perfect lesion-deficit inference with high sensitivity and specificity. Have the problems thus been solved and does multivariate inference represent the ultimate method for lesion-deficit inference?

The work nicely illustrates that multivariate models and inference methods based on them are required to properly represent multidimensional phenomena such as the interaction of brain regions in networks. The ability to represent these is an obvious strength of the multivariate approaches. We also agree that no simple methodological tweak—such as control for lesion size—can properly address the limitations of univariate inference. However, we also feel compelled to dampen the optimism concerning multivariate inference. Specifically, we see two important aspects that we need to consider to fully understand the challenges that we face in brain pathology-deficit inference.

## Brain-deficit relationships are not necessarily highly sparse

**Figure 1:**
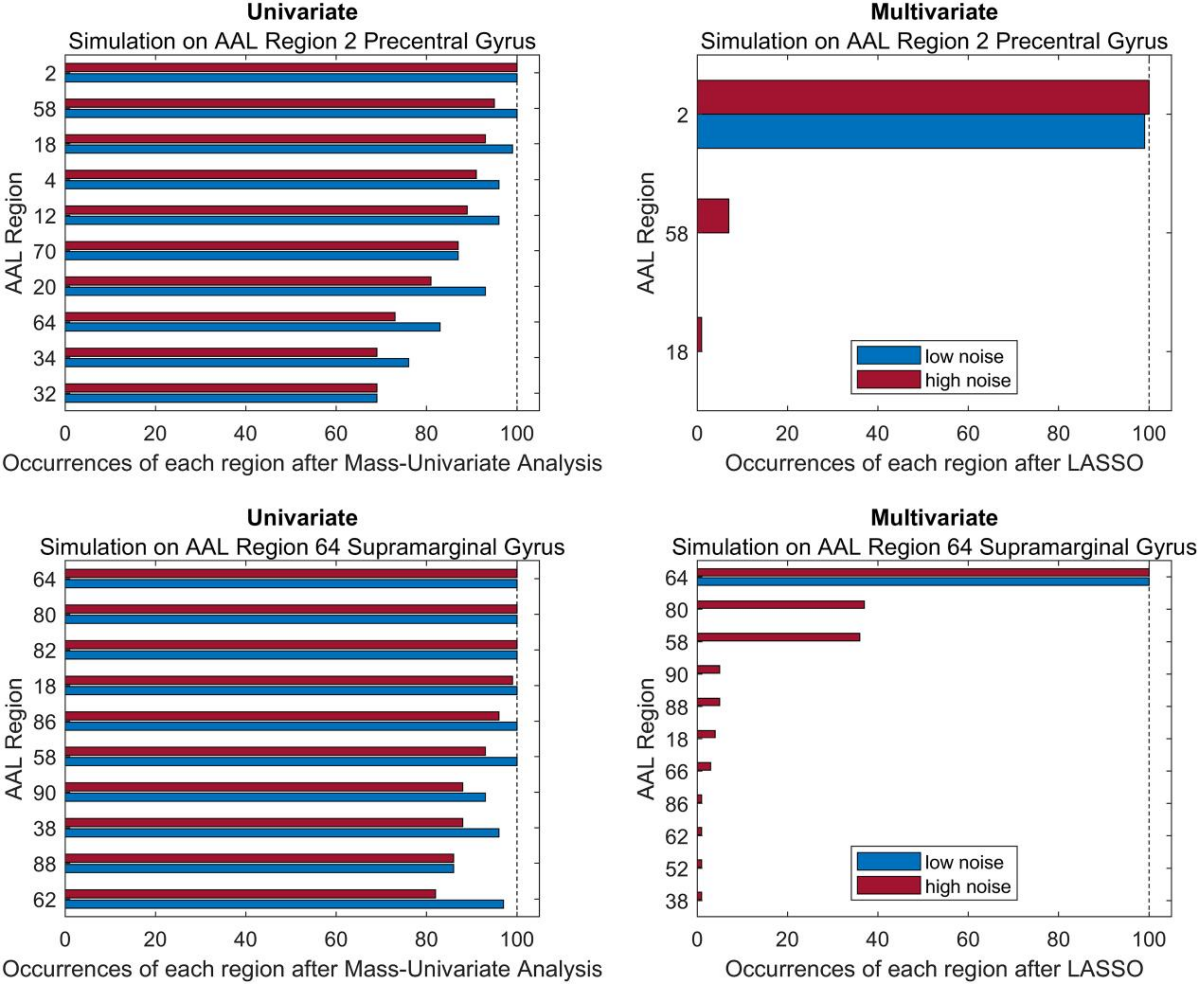
**Univariate and multivariate inference with Lasso in a simulation setting.** Results of the repeated application of univariate mapping or multivariate Lasso regression in subsamples of 120 out of 180 patients for simulated deficits. The true neural correlates of the mapped deficits were either the precentral gyrus (AAL region 2, first simulation) or the supramarginal gyrus (AAL region 64, second simulation). For univariate analyses, only the most often implicated regions are shown. In summary, (i) the results in univariate analyses are highly unspecific with many false positives; and (ii) multivariate Lasso regression almost perfectly identifies the true neural correlates of a simulated deficit with both high sensitivity and specificity.

Any multivariate inference method that creates a highly sparse model might excel in a simulation setting with simple uni- or bi-regional neural correlates, but might not perform well in real data. To illustrate this, we consulted a data set of 180 stroke patients taken from a recent study on post-stroke cognitive impairment.^7^ For all patients, we obtained normalized lesion masks and neuropsychological assessments with continuous measures of selective attention and visuoconstructive ability (for details, see Gallucci *et al*.^7^). In the first analysis, we performed a simple simulation where a deficit was caused by damage to only a single region, as also included in the study by Zavaglia and colleagues. We did so separately for two regions, i.e. performed two different simulations. We simulated a deficit equivalent to the proportion of lesioned voxels either in the precentral gyrus or the supramarginal gyrus. We defined brain regions by the Automatic Anatomical Labelling (AAL) atlas included in MRIcron (https://www.nitrc.org/projects/mricron). On top, we added random normal noise based on the standard deviation (SD) of the proportions of lesioned voxels per region. We set the standard deviation of the normal noise either to 0.2 ∗ SD (low noise) or to 1.0 ∗ SD (high noise). For 100 times, we repeatedly drew a random sample of 120 subjects (i.e. two-thirds of the total sample) and mapped the neural correlates of the simulated deficit across all 116 regions included in the brain atlas. We used multivariate Lasso regression with 5-fold cross-validation and univariate repeated Pearson correlation analysis with Bonferroni correction. The Lasso (least absolute shrinkage and selection operator) is a prominent algorithm for creating sparse computational models from high-dimensional data. The outcome measure was the number of occurrences of each region as a neural correlate across the 100 repetitions of the analysis, i.e. the number of times each of the 116 atlas regions (i) retained a non-zero coefficient in the Lasso regression; or (ii) was significant at a corrected *P* = 0.05 in the univariate analysis. The results (Fig. 1) replicated the findings by Zavaglia and colleagues: univariate analyses were drastically unspecific as they consistently implicated a large number of regions beyond the single ground truth region. Lasso regression, on the other hand, performed almost perfectly in the low noise condition, and still very well in the high noise condition where it occasionally implicated very few other regions. Is multivariate inference with lasso regression thus a strong method for lesion-deficit inference?

**Figure 2:**
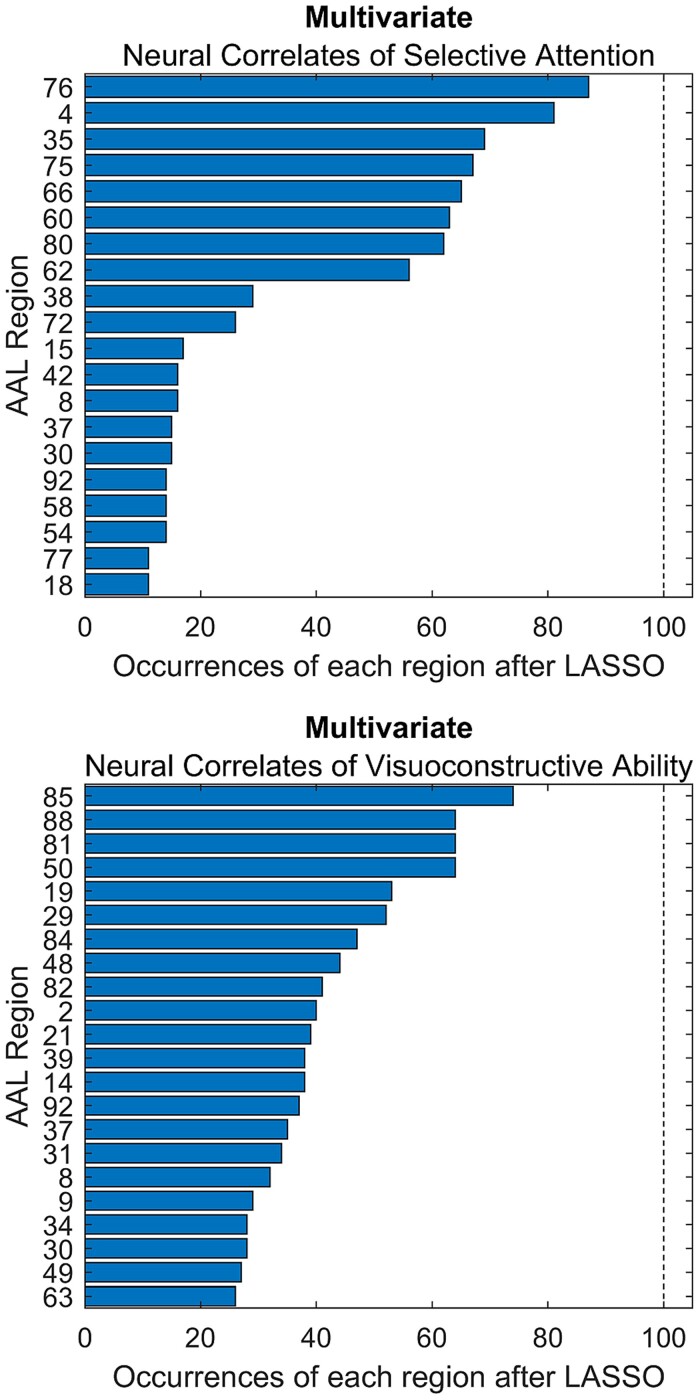
**Multivariate inference with Lasso in real-world data.** Results of the repeated application of Lasso in subsamples of 120 out of 180 patients for selective visual attention and visuoconstructive ability. Only regions that were found at least 10 (selective attention) or 25 (visuoconstructive ability) times in 100 repetitions are reported. In summary, the regions found by multivariate analysis with the lasso operator were highly heterogeneous across repetitions and a large number of regions were repeatedly implicated.

To elaborate on this, we also used Lasso regression to model the two real-world deficits. Here, the stability of results looks markedly different than in the simulation setting (Fig. 2). The large set of implicated regions varied drastically across repetitions—no even remotely stable sparse model could be found to explain the neuropsychological deficits. One explanation is that the current sample sizes may simply be too low to compute a stable multivariate model. Another explanation is that the neural correlates of a cognitive function cannot be properly represented by a highly sparse model. In contrast to the simulation, we may be unlikely to encounter a real cognitive deficit that can be almost perfectly predicted from damage to only a single region. Deficits may originate from damage to numerous features and can be influenced by a variety of secondary clinical or demographic factors. Additionally, their assessment is often notoriously noisy. And even if the neural correlates of a deficit were in principal representable by a highly sparse model, the chosen imaging variables should closely represent those. If we try to model a deficit caused by disconnection of a white matter fibre bundle by the lesion load of grey matter regions, the algorithm may be bound to fail. Last, another issue may cause highly unstable models, which leads us to the next major point.

## Lesion-deficit inference is not only a problem of multi-dimensionality but also of causality

There is a misconception about what multivariate inference can accomplish when mapping brain pathology. It was postulated that multivariate inference overcomes two limitations of univariate methods at the same time: (i) the inability to represent multidimensional and interacting phenomena; and (ii) the inability to account for typical associations between brain imaging features indicating pathology.^2,3^ The first point may not only be intuitively convincing in theory but has also been supported by numerous studies.^2,4^ However, by reframing the second point, a glaring error in reasoning can be identified.

Human pathological brain imaging data are (i) observational data; and (ii) an epitome of multicollinearity. For stroke lesions, the associations between imaging features and thereby the multicollinearity are so extreme that the lesion status of over 900 000 voxels can be almost completely explained by a few dozen principal components.^8^ The ideal lesion-deficit inference method should now be able to infer causality: perturbation of which features ‘caused’ the deficit?

In inferential analysis with low-dimensional data, the problem of multicollinearity is well-known. For example, imagine multiple linear regression to gauge the possible effects of a few clinical or demographic variables on a clinical outcome. If the variance inflation factor suggests high multicollinearity, the regression model is commonly considered inappropriate for this purpose. The fact that the regression is multivariate is not seen as a saving factor. In general, the inference of causal relationships is already a challenge in low-dimensional observational data. This challenge is the focus of the field of ‘causal inference’. Machine learning and deep learning explicitly do not provide causal inference, as they operate in purely associational mode.^9^ The challenge of causal inference is amplified in high-dimensional data—it is no coincidence that there are fields such as ‘Causal inference in big data’ or ‘Causal machine learning’, which are differentiated from classic machine learning.^10^

In conclusion, multivariate inference techniques represent a significant step forward for lesion-deficit inference by capturing the multidimensional and interacting organization of the human brain. However, they still face challenges in inferring causality in highly multicollinear data, similar to univariate methods. This is probably also the reason why some simulation studies have found no consistent advantage of multivariate over univariate methods.^5,6^ Finally, to come back to the previous point on the potential limitations of sparse models: if perturbation of one out of many highly correlating features causes a deficit, then all of these features can predict the deficit. Non-zero coefficients may then be exchangeable between these features, and many different sparse models can explain the data. In other words, a model may identify a feature that is associated with an outcome, but which is not necessarily the cause of it. Thereby, it fails the original intention behind lesion-deficit inference.

## Data Availability

The MATLAB scripts underlying the simulation are available on Mendeley data at doi:10.17632/xjyr3f2v5v.1.

## References

[1] Noble S, Curtiss J, Pessoa L, Scheinost D. The tip of the iceberg: A call to embrace anti-localizationism in human neuroscience research. Imaging Neurosci. 2024;2:1–10. 10.1162/imag_a_00138 PMC12247580 40740614

[2] Zavaglia M, Malherbe C, Schlaadt S, Nachev P, Hilgetag CC. Ground truth validation of uni- and multivariate lesion inference approaches. Brain Commun. 2024. Advance Access published on July 26, 2024, doi:10.1093/braincomms/fcae251 PMC11406464 39291162

[3] Mah YH, Husain M, Rees G, Nachev P. Human brain lesion-deficit inference remapped. Brain. 2014;137(9):2522–2531. 24974384 10.1093/brain/awu164 PMC4132645

[4] Zhang Y, Kimberg DY, Coslett HB, Schwartz MF, Wang Z. Multivariate lesion-symptom mapping using support vector regression. Hum Brain Mapp. 2014;35(12):5861–5876. 25044213 10.1002/hbm.22590 PMC4213345

[5] Sperber C . Rethinking causality and data complexity in brain lesion-behaviour inference and its implications for lesion-behaviour modelling. Cortex. 2020;126:49–62. 32062142 10.1016/j.cortex.2020.01.004

[6] Ivanova MV, Herron TJ, Dronkers NF, Baldo JV. An empirical comparison of univariate versus multivariate methods for the analysis of brain–behavior mapping. Hum Brain Mapp. 2021;42(4):1070–1101. 33216425 10.1002/hbm.25278 PMC7856656

[7] Gallucci L, Sperber C, Guggisberg AG, et al Post-stroke cognitive impairment remains highly prevalent and disabling despite state-of-the-art stroke treatment. Int J Stroke. 2024. Advance Access published on March 01, 2024, doi:10.1177/17474930241238637 38425239

[8] Salvalaggio A, de Filippo De Grazia M, Zorzi M, de Schotten MT, Corbetta M. Post-stroke deficit prediction from lesion and indirect structural and functional disconnection. Brain. 2020;143(7):2173–2188. 32572442 10.1093/brain/awaa156 PMC7363494

[9] Pearl J, Mackenzie D. The book of why. Basic Books; 2018.

[10] Feuerriegel S, Frauen D, Melnychuk V, et al Causal machine learning for predicting treatment outcomes. Nat Med. 2024;30(4):958–968. 38641741 10.1038/s41591-024-02902-1

